# Mimicking Anti-Viruses with Machine Learning and Entropy Profiles

**DOI:** 10.3390/e21050513

**Published:** 2019-05-21

**Authors:** Héctor D. Menéndez, José Luis Llorente

**Affiliations:** 1 Computer Science Department, University College London, London WC1E 6BT, UK; 2 Hoffmann-La Roche, 28027 Madrid, Spain

**Keywords:** anti-virus, classification, malware, mimicking, mimickAV, entropy profiles

## Abstract

The quality of anti-virus software relies on simple patterns extracted from binary files. Although these patterns have proven to work on detecting the specifics of software, they are extremely sensitive to concealment strategies, such as polymorphism or metamorphism. These limitations also make anti-virus software predictable, creating a security breach. Any black hat with enough information about the anti-virus behaviour can make its own copy of the software, without any access to the original implementation or database. In this work, we show how this is indeed possible by combining entropy patterns with classification algorithms. Our results, applied to 57 different anti-virus engines, show that we can mimic their behaviour with an accuracy close to 98% in the best case and 75% in the worst, applied on Windows’ disk resident malware.

## 1. Introduction

Malware is proliferating and growing exponentially every year, especially in the current software markets [1]. This is due to several different reasons, some of which relate to Moore’s law, which states the exponential growing tendency of technology, and others to the cybersecurity arms race. Emerging technologies, such as the internet of things [2], create new vulnerabilities that are normally easily exploited. The cybersecurity arms race also focuses on new steps on already known systems. Final users are normally ignorant to the infections on their machines, relying on anti-viruses to deal with these threats. However, the reliability of anti-viruses is not the strongest line of defence, although several anti-virus companies are making strong efforts to deal with malware in a timely fashion.

Detection of malware relies on static and dynamic software analysis [3]. On the former, the software is analysed with no execution. On the latter, the software is executed and its traces are analysed. Depending on the analysis goals and strategies, this can be extremely time consuming. Normally, dynamic analysis requires longer time to detect malicious behaviours than static analysis, as the malware needs to activate it during execution, which might depend on potential enviromental conditions that trigger it [4]. The highest and most costly level of analysis is manual reverse engineering, which is normally targeted when a triage process puts the malware at the top level of threat. Nevertheless, different works have strong caveats on static analysis, providing malicious concealment via metamorphism or polymorphism, and dynamic analysis via red pills. Besides, modern methods can even attack the triage process, shaping the malicious piece to look as benign as possible using machine learning [5].

One of the most interesting steps forward of the anti-virus companies is VirusTotal [6], a platform where anybody can submit a piece of software that will be analysed by several different anti-virus engines. This mechanism helps to understand not only which anti-viruses detect malicious behaviours, but it also provides information related to automatic dynamic and static analysis of the binary. The usefulness of VirusTotal makes it a good honey-pot for black hats, helping anti-virus companies to collect new malware pieces that are in the wild. However, does a black hat really need to use an anti-virus to evaluate whether their malware is detected or not? This question motivates our work.

Considering that several anti-viruses normally perform statistical analysis using features extracted from the binary file, such as: n-grams, entropy, opcodes, signatures, size, imports section, or in specific kinds of malware like Android malware, permissions and intent actions, among others [7], we want to know how accurately we can predict the outcome of an anti-virus given a piece of software. Mimicking anti-viruses supposes that a black hat might not need to use it, in order to evaluate the detection of their malware or malware variants, but instead they can use their own predictors. These copies can be created based on the information already known from the anti-virus by using machine learning methods. The mimicking process only needs two things: a proper set of features, representative enough to extract strong patterns from the detection of malware, and a proper learning system.

As this work pretends to create a general approach that can work with any kind of binary file, we apply entropy profiles as features of the binaries [8]. An entropy profile considers the binary representation of a file, divides it into chunks, calculates the Shannon entropy of these chunks, and aggregates them, forming an entropy sequence called the entropy profile. These entropy profiles have shown a strong accuracy for deciding whether a piece of software is malware or not, even when the piece of malware is using metamorphic or polymorphic/packing-based concealment [9]. Their strength comes from the entropy variations on the binary files. These variations are similar among different variants of the same malware or different concealments using the same or similar strategies [8,9,10]. These variations, or signals, are relevant patterns to distinguish different kinds of binaries. Here, we want to evaluate if these patterns can lead to learn the anti-virus itself, by learning how it behaves under different entropy variations.

For this purpose, we have created a whole framework, called MimickAV, based on applying different classification algorithms to reproduce the behaviour of several anti-viruses. These classifiers have been chosen from different machine learning paradigms, in order to understand which statistical models are better for approximating the behaviour of anti-virus engines. Our experiments focus on disk resident malware, concretely Windows PE32, extracted from VirusShare. We also collect benign-ware to balance the detection. To make sure that the concealment strategies do not affect the detection quality, we independently evaluate packed and non-packed malware and, after, we evaluate them in an aggregated way. The results show that we have a strong accuracy mimicking the anti-virus engines from VirusTotal, especially when we apply classifiers based on boosting. The classifiers can mimic every anti-virus with an accuracy up to 98%. Our main contributions can be summarized as follows:

- We introduce MimickAV, the first general purpose method that can mimic almost any current commercial anti-virus engine by using entropy profiles, a feature that every single binary file has (Section 2).
- We apply this methodology using 20 state of the art machine learning classifiers from 10 different paradigms with the aim of mimicking 57 commercial anti-viruses from VirusTotal (Section 3).
- From the 20 machine learning algorithms, we proved that boosting is the strongest, reaching an accuracy between 96% and 98% for packed malware, 93% and 96% on mix malware, and 85% and 96% on non-packed malware (Section 4.1 and Section 4.2).
- We show which anti-virus are more resistant to our method, and which specific concealment strategies make harder to apply entropy to mimic the anti-viruses responses (Section 4.3 and Section 4.4).

## 2. MimickAV: Mimicking Anti-Virus Software

Our goal is to show how to predict the behaviour of anti-virus software by using their own detection decision. To reach this goal, we introduce MimickAV, a framework that combines entropy profiles with different machine learning methods in order to mimic anti-viruses. As the anti-virus normally uses features extracted from the binary file itself, we will use an aggregation of these features in the form of an entropy profile (Section 2.1). After, we explain the classifiers applied in this process (Section 2.2).

### 2.1. Entropy Profiles

The process of extracting an entropy profile is based on Sorokin’s structural entropy method [8]. For a detailed description we reference Section 2 of [9], the following summarizes this process. The main three steps are:

1. Partition the file in chunks, i.e., small parts of the same size, and calculate their entropy to generate an entropy sequence.
2. Shrink or extend the entropy sequence to a specific length, which normalizes the approach to different file lengths.
3. Clean the noise of the sequence using the Harr wavelet. The cleaned sequence is the entropy profile.

Consider a program P as a binary string of size $s_{P}$, and the chuck size $s_{c}$, both sizes in bytes. We partition P in $s_{P}/s_{c}$ chunks, starting from the beginning of the binary string. Suppose $C=\{c_{1},\ldots ,c_{N}\}$ as the set of all chunks (*N* chunks) after partitioning. Following the work of Sorokin [8] on entropy profiles and subsequent works [9], we consider the entropy at byte granularity, therefore we use the chunk string as a byte string, and calculate the probability of every byte inside the chunk to measure the Shannon entropy. The Shannon entropy, which measures the levels of randomness on data, for a specific chunk $c_{i}$ is computed as:

(1) $$H\left(c_{i}\right)=-\sum_{b\in B}p\left(b\right)\log_{2}p\left(b\right),$$

where *B* is the set of all possible bytes (*b*) in the chunk string. This process is applied to every chunk to generate the entropy sequence, denoted by $H\left(C\right)=\{H\left(c_{1}\right),\ldots ,H\left(c_{N}\right)\}$. Nevertheless, as the chunk size is fixed, files of different sizes would have different cardinality for H(C), therefore, we need to normalize this value. The normalization is related with the cleaning process based on wavelets, used to focus the entropy sequence only on the main entropy variations. This normalization process will select specific chunks form H(C) to fit a specific size. This process is similar to time series comparison, where time series of different lengths are normalized to have the same length. We need to select a number of chunks that is a power of 2. Let $M=2^{a}$ be the final number of chunks, the process selects the first and last chunks (which are normally special sections of the file [9]), and, then, selects each chunk by index, with an increment of inc=(N−1)/(M−1). We consider $X=x_{1},\ldots ,x_{M}$ the entropy sequence after the reduction/enlargement process.

Finally, the cleaning process eliminates noise from the entropy sequence and keeps only the relevant entropy variations. This process extracts the Harr wavelet from the entropy profile. The Harr wavelet coefficients are divided into scales, where the next scale is calculated recursively from the previous one, starting from the entropy profile. Each scale is divided in two parts (this is the main reason of forcing the sequence length to be $2^{a}$). The first part is called the scale coefficients ($s_{i}$), and the second is called the detail coefficients ($d_{i}$). A scale coefficient is calculated from the previous scale as:

(2) $$s_{i}^{1}=\frac{1}{\sqrt{2}}\left(x_{i}+x_{i+1}\right),\;\;\;s_{i}^{a}=\frac{1}{\sqrt{2}}\left(s_{i}^{a-1}+s_{i+1}^{a-1}\right),\;\;\;a>1,$$

while a detail coefficient is calculated by:

(3) $$d_{i}^{1}=\frac{1}{\sqrt{2}}\left(x_{i}-x_{i+1}\right),\;\;\;d_{i}^{a}=\frac{1}{\sqrt{2}}\left(s_{i}^{a-1}-s_{i+1}^{a-1}\right),\;\;\;a>1,$$

Suppose that we set a=2, therefore the wavelet has up to two scales, starting from the entropy sequence:

$$\begin{array}{c} \left(x_{1},x_{2},x_{3},x_{4}\right)\\ ↑↓\\ \left(s_{1}^{1},s_{2}^{1}\;|\;d_{1}^{1},d_{2}^{1}\right)\\ ↑↓\\ \left(s_{1}^{2}\;|\;d_{1}^{2},d_{1}^{1},d_{2}^{1}\right) \end{array}$$

Our cleaning process works at the last scale, in this example $\left(s_{1}^{2}\;|\;d_{1}^{2},d_{1}^{1},d_{2}^{1}\right)$. It sets a threshold τ on the coefficients and set to 0 every value under this threshold. This removes noise from the wavelet, keeping only the relevant variations on the entropy sequence after its reconstruction. The reconstruction of the scales uses the inverse process:

(4) $$\begin{array}{c} x_{k}=\frac{1}{\sqrt{2}}\left(s_{k}^{1}+d_{k}^{1}\right),\;\;\;s_{k}^{a}=\frac{1}{\sqrt{2}}\left(s_{k}^{a+1}+d_{k}^{a+1}\right),\;\;\;a>0,\\ x_{k+1}=\frac{1}{\sqrt{2}}\left(s_{k}^{1}-d_{k}^{1}\right),\;\;\;s_{k+1}^{a}=\frac{1}{\sqrt{2}}\left(s_{k}^{a+1}-d_{k}^{a+1}\right),\;\;\;a>0, \end{array}$$

After the reconstruction, the values of *X* are smoother. We consider this reconstruction as the entropy profile of the binary that will provide the features for the classification process. For each program, we will extract its entropy profile. It is important to remark that after the reconstruction, the Harr wavelet provides a clean signal for a machine learning algorithm.

### 2.2. Classification

In order to mimic the anti-virus behaviour, we leverage classification algorithms. As we have no information on how the anti-viruses are detecting malware, we can hardly approximate which classification method would be more suitable for mimicking them. Therefore, we consider different classifier families:

- Tree-based classification [11]: these classifiers divide the data in a linear fashion, defining a tree of decisions on their features. This division is chosen by a metric, normally the entropy.
- K-nearest neighbourhood [12]: the k-NN algorithm decides the classification on an instance based on its k-nearest neighbours. Normally, this decision is the majority of the neighbours classes.
- Support Vector Machines [13]: SVM creates a hyperplane to separate the data into classes. This hyperplane maximizes its margin with the frontier of each data class, defined by specific instances called support vectors. It normally applies kernels to work with non-linear separations.
- Rules-based classification [14]: these classifiers create a set of rules that aim to generalize the classification decision of each instance based on its features.
- Naïve Bayes [15]: this classifier learns a probability distribution for each feature, considering each one independent, and applies Bayesian probability on the features distribution to decide when an instance is assigned to a class.
- Random Forest [16]: this method combines different tree-based classifiers into a voting system. Normally the algorithm splits the feature space and train each tree in different combinations of features.
- Boosting [17]: following the same logic than random forest, this algorithm is a multi-learning approach where several weak classifiers are combined to learn different areas of the feature space and aggregate them to provide a final classification decision.
- Generalised Linear models [18]: this algorithm generalizes linear regression to different probability distributions. The aim is to separate the data by learning the parameters of specific probability distributions that work as an estimator.
- Artificial Neural Networks [19]: an artificial neural network is a hierarchical structure of nodes connected by layers, where the top one is the input and the bottom one is the output. Between these layers there are hidden layers that aim to reproduce the behaviour of the human brain.
- Deep Learning [20]: deep learning algorithms are a generalization of neural network where the algorithm combines unsupervised learning and supervised learning in its hidden layers.

These families have been selected for two reasons. First, they cover a huge spectrum of different machine learning paradigms, and second, they use different statistical models that can be adapted to the behaviour of specific anti-viruses.

## 3. Experimental Setup

MimickAv requires a detailed evaluation in terms of performance and accuracy. Section 3.1 explains the steps that we have followed on its evaluation. Section 3.2 details the datasets used. Section 3.3 shows the anti-virus engines in which we evaluated MimickAV, and Section 3.4 details the set up for the classifiers.

### 3.1. Research Goals

Our system aims to mimic the behaviour of different anti-virus engines. In order to be able to generalise, we need to understand the abilities of the system for different kinds of malware and different anti-viruses. Although the study can be extended to Android, JavaScript, and PDF malware, among others, we focused on disk resident malware as our proof of concept, concretely Windows PE32 malware. We also consider packed malware during our experimentation as packers apply compression and encryption, directly affecting the entropy of the files. It is important to remark that the concept of entropy profile can be applied to any type of binary or text file.

In order to understand how our methodology can reach our research goals, we have divided our experiments in order to answer the following research questions.

**RQ 1.** *What is the accuracy and performance of MimickAV when it is applied to packed, non-packed and mixed binary files?*

To answer this RQ we need to collect specific malware and benign-ware which are either packed and non-packed. Then we will apply our learning process with different classifiers in order to evaluate which one is more accurate during the mimicking process.

**RQ 2.***What is the precision of MimickAV when it is applied to the different kinds of binaries?*   

One of our main concerns is to ensure that we have the lowest possible number of false negatives, i.e., none of the malware that we pass to the system is falsely considered as benign in our mimicking process. For this reason, we also measure the Receiver Operating Characteristic (ROC) curve, but we focus it on the false negatives instead of the traditional false positives rate.

**RQ 3.***Which specific packing systems are more resistant to MimickAV?*   

We aim to understand which specific packers make harder to predict the behaviour of the anti-viruses, based on our entropy features.

**RQ 4.** *Which anti-viruses are more, and which ones are less resistant to our mimicking process?*

We rank the abilities of the whole set of anti-virus where we operate to measure those that are more resistant in general.

The following section explains the data that will be applied for answering to these research questions and how these data have been extracted and processed.

### 3.2. Evaluation Data

Our evaluation of the mimicking process is focused on Windows resident malware. We have collected malware data from VirusShare. From this source, we collected packed and non-packed malware. In order to balance these datasets with benign-ware, we have also collected fresh packed and non-packed benign-ware files.

VirusShare (http://virusshare.com) is a storage webpage for malware data containing several different kinds of malware from different periods. For these experiments, we focused our extraction process on disk resident malware for Windows. To identify the packed malware we applied Yara (http://yara.readthedocs.org) and the set of rules applied for identifying packed software from the YaraRules project (http://yararules.com/). Yara identified 10,000 malware pieces that use known packers and 6,000 that were not packed. The number of packers that this dataset contains is around 70 from several different families. Dividing them into families, we identify 7 predominant ones (Table 1). The software was collected between 2015 and 2016. As we do not have a ground truth for the malware families, we used the Avast anti-virus’ reports which provide information about the detection. Avast detects 364 different families in the packed corpus and 266 in the non-packed corpus. The packed corpus’ size ranges from 4 Kb to 32 Mb with an average of 515 Kb, and the non-packed corpus’ size ranges from 4 K to 32 Mb with an average of 747 Kb.

In order to balance these data, we extracted a set of disk resident benign-ware for Windows from download.com. This benign-ware is divided into two sets: packed and non-packed. The benign packers are also described in Table 1. We extract 2,000 samples for each set. The packed benign-ware was also identified using Yara. The packed corpus’ size ranges from 20 Kb to 9 Mb with an average of 2 Mb, while the non-packed benign-ware ranges from 4 Kb to 26 Mb with an average of 3 Mb.

For our experiments we create different datasets to evaluate the mimicking abilities of our methodology depending on the nature of the data. We make sure that the classes (malware and benign-ware) are balanced to avoid imbalance problems related to the machine learning algorithms, as those mentioned in [21]. These datasets are:

- Pck data. Composed of 2000 samples from VirusShare selected uniformly at random without repetition from the 10,000 described in Table 1, and the 2000 samples from the packed benign-ware.
- NPck data. Composed of 2000 samples from the non-packed data of VirusShare selected uniformly at random without repetition from the 6000 malware, and the 2000 samples from the non-packed benign-ware.
- Mix data. It is a mix of the previous dataset with the aim of generalising the mimicking abilities of the system. These data contains 4000 samples, 1/2 of packed malware and benign-ware, and 1/2 non-packed malware and benign-ware. It aggregates the Pck and NPck data.

For each sample of the data, we will extract the entropy profile as explained in Section 2.1. The application of the Harr wavelet will de-noise the entropy profiles providing clean signals for the machine learning algorithms (step 3, Section 2.1).

### 3.3. Anti-Virus Selection

The system has been evaluated using several anti-virus engines. These anti-viruses are commercial versions which are available in VirusTotal. VirusTotal is a malware analysis service that allows to upload a binary file and runs several anti-virus engines to it, in order to evaluate whether a binary file is malicious or not. There are 82 different anti-viruses on VirusTotal at the moment. For our experiments, we submit all our binary files to the system. We collect only those reports that specify which anti-virus has detected the malware. In the case of the benign-ware, all anti-virus provided a negative detection. During the detection process, not all of the 82 anti-virus are activated by VirusTotal. To guarantee enough data for the training process, we filter those anti-viruses that failed to activate more than 500 times with our malware. The final number of anti-viruses that we applied for the experiments is 57. For each anti-virus we will select the previous datasets (PcK, NPck, and Mix) based on its detections. The sampling process will select first among the detected malware. If there is not enough malware, it will complement the information with non-detected malware until it reaches the 2,000 malicious samples. The other samples will be the corresponding benign-ware.

### 3.4. Classification Algorithms

For the classification process we have applied 20 different classifiers, organized by the MLR package (https://cran.r-project.org/web/packages/mlr/index.html) [22] of the R-project. This package simplifies the interface with different machine learning algorithms. In our case we have chosen classifiers from different families in order to evaluate which kind of classifier has better performance (Section 2.2). For each classifier family, we have chosen, at least, one classifier, but we gave priority to the best state of the art implementations. The specific list of algorithms and packages is the following:

- Tree-based classifiers: we have applied the classical J48 [11], from the RWeka package [23], and its specialization applying separate-and-conquer algorithms, PART [24]. We have also applied the recursive partition algorithm, rpart, from the homonym package [25].
- K-nearest neighbours: for k-nearest neighbours, we have applied the classical knn implementation from R [12], and the Instance-based learning classifier (Ibk) from RWeka [26].
- Support Vector Machines: we applied the classical svm implementation from the e1071 package [27], and the implementation of kernlab which also optimize the kernel parameters, ksvm [28].
- Rules-based: we applied the implementation of the classical Repeated Incremental Pruning to Produce Error Reduction (RIPPER) algorithm [14] from the RWeka package (JRip), and the One Rule (OneR) algorithm [29] that generates one rule for each predictor in the data.
- Naïve Bayes: we apply the classical naiveBayes algorithm from package e1071 [15].
- Random Forest: we applied one of the current state of the art implementations from the H2O package: h2o.rForest [16].
- Boosting: since boosting is one of the current strongest classifiers for entropy profile discrimination [10], we applied several versions of it. First, we use the original boosting [17], that we used in our previous work [9] leveraging rpart from the multi-learning approach. Then, we apply adaptive boosting (ada) [30], from the homonym package [31], which adapts the learners weight in favour of those instances misclassified by previous classifiers. We also applied two modern versions of gradient boosting [32], an adaptation of adaptive boosting with the ability of optimizing a cost function. We consider the implementation of gbm package and the parallel version of the algorithm from the H2O package (h2o.gbm), which also includes an automatic detection system for different loss functions. Finally, we consider the extreme gradient boosting algorithm, xgboost, which reduces feature splits in order to reduce the search space [33].
- Generalized Linear Model: for GLM we use the implementation from H2O package, h2o.glm [18].
- Deep Learning: for deep learning, we also apply the H2O implementation of deep learning algorithms, h2o.deepL [20].
- Neural Network: for neural networks, we applied the classical nnet implementation from the homonym R-package [19] and Learning Vector Quantization [34], lvq1, from the R-project core, whose interpretation is easier than neural networks.

The classifiers parameters are selected by default. The parameters for the entropy profiles were chosen following the same criteria as [9]. These parameters are the following: the chunk size is 256 bytes, the length of the entropy profile is 512 coefficients (hence, the scale is 9), and the threshold for the cleaning process using wavelets is 0.5. The entropy profiles and the implementation of the algorithms is publicly available in: https://github.com/hdg7/MimickAV.

## 4. Experiments

In order to evaluate MimickAV with respect to our research goals (Section 3.1), we start doing a global analysis using every available classifiers described in Section 3.4, and each of the 57 anti-virus engines. Then, we focus the analysis on the best classifier to evaluate their ability to reduce false negatives, our target with MimickAV (Section 4.2). As the results of MimickAV can be altered by different packer systems, Section 4.3 increments our classification granularity to check which packers are more resistant during the mimicking process. Finally, we provide an analysis on the most and less resistant anti-viruses to the mimicking process in Section 4.4. Every classifier is trained with 2/3 of the data and tested with 1/3. Every result reported is on the test data.

### 4.1. Performance of MimickAV

**Figure 1:**
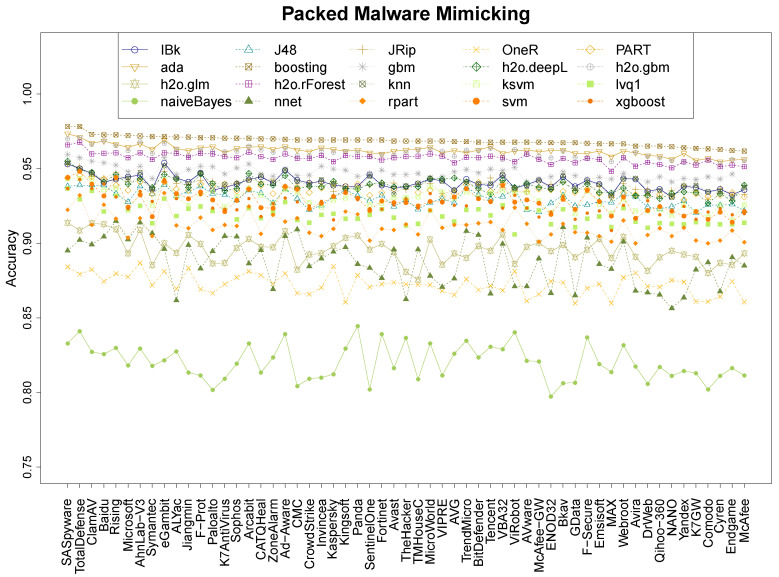
Accuracy of the 20 classifiers on the 57 anti-viruses for the Pck data.

**Figure 2:**
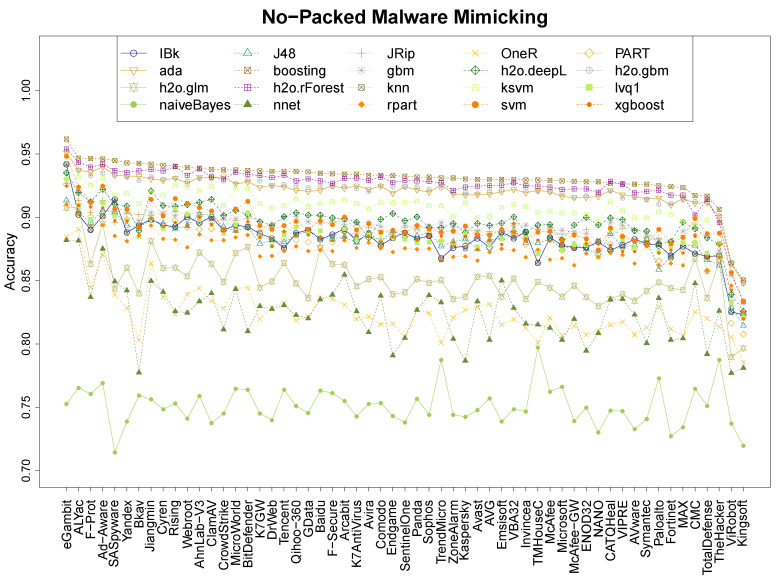
Accuracy of the 20 classifiers on the 57 anti-viruses for the NPck data.

**Figure 3:**
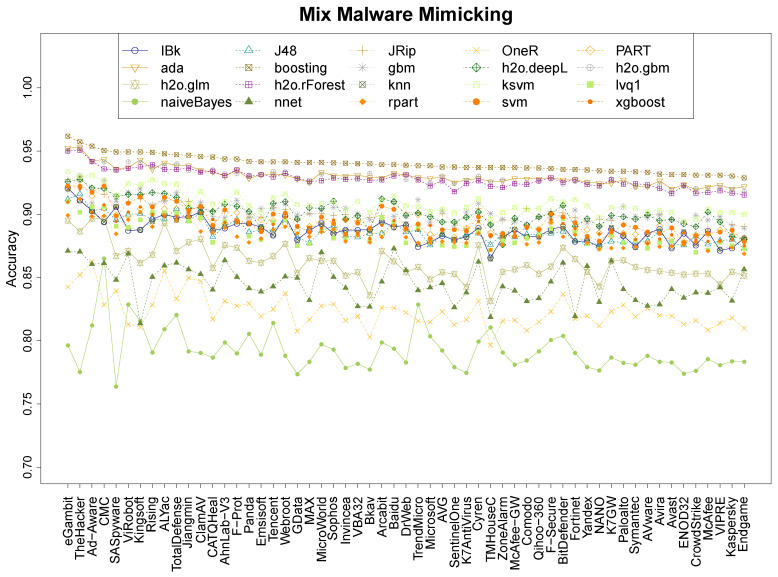
Accuracy of the 20 classifiers on the 57 anti-viruses for the Mix data.

Our experiments measure the quality of MimickAv learning the output of different anti-viruses. This first experiment focuses on understanding its prediction abilities on the 57 anti-viruses of VirusTotal. Figure 1 shows the median results over 20 repetitions for the 20 classifiers on packed malware, Figure 2 shows the equivalent results on non-packed malware and Figure 3 shows the results on the mix data. For clarification purposes, the most mimicked anti-viruses (and less) and the best/worse classification techniques of the mimicking process are also shown in Table 2. The figures show that the classifiers follow a ranking tendency, especially the top four, where those who are the best at learning a specific anti-virus are also the best learning any of them. This is the case for booting, ada, h2o.gbm, and h2o.rForest. This tendency does not apply for the following classifiers in the quality ranking, where the classifiers mix. Nevertheless, the hierarchy of classification quality is the same for every dataset, where boosting is always the predominant and the other 3 top algorithms keep similar results. In every single dataset, the naiveBayes classifier obtains the worst results.

In terms of mimicking ranges, we can see that the global ranges are different between the three datasets. The packed dataset has the best accuracy range, between 96.2% and 97.8% (Table 2, boosting) of accuracy for the best classifier, and between 79.7% and 84.4% of accuracy for the worst (Table 2, NBayes). The non-packed dataset has the worst results, between 85 and 96.2% of accuracy for the best classifier (Table 2, boosting), and 71.4% and 79.7% for the worst (Table 2, NBayes). The mix dataset has an intermediate range: the best is between 92.9% and 96.2% (Table 2, boosting), and the worst between 76.4% and 86.5% (Table 2, NBayes). The results suggest that the packers tend to leave clearer entropy patterns on the binary that those found on non-packed files, as different files tend to use similar packers (this is also analysed in Section 4.3).

Research question 1 asked about the accuracy performance of MimickAV. It can, indeed, mimic the behaviour of any anti-virus of VirusTotal reaching an accuracy up to 98% on packed data, and 96% on non-packed and mix data, using a boosting classifier.

### 4.2. Precision of MimickAV

The main goal of MimickAV is to understand how an anti-virus is predicting malware in order to provide an imitation of this prediction. In this black hat scenario, it is important to make sure that MimickAV produces the lowest possible number of false negatives, as a false negative in this imitation process is a variant that will be detected in the wild. Therefore, in this context, we aim to study the negative predictive value, defined as the rate between true negatives and total negatives. For this reason, we studied the inverse ROC curve, focusing on the trade off between false and true negatives depending on the classifier prediction threshold.

**Figure 4:**
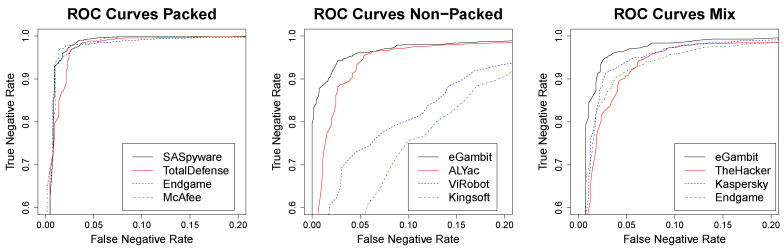
Boosting results of the inverse ROC curves (true vs false negative rates) for the best and worst anti-viruses.

Based on the results of Table 2, we choose the best classifier (boosting) to evaluate the limits on the AV imitation process. Table 3 shows the specific accuracy results for the top and bottom anti-viruses. The table shows the evolution of true and false negatives rate, as we move the detection threshold from 1 to 0, and compare these two values. It also divides the comparisons on the three different datasets considered: Pck, NPck, and Mix. These results are also graphically represented in Figure 4. We can see that for both, the PcK data and the Mix data, all the anti-viruses have similar results in the curve evolution, however, in the case of NPck, there is a representative difference between the best and the worse.

Focusing on the evolution of the threshold, when the curve reaches 0.01 false negatives rate, every anti-virus of the PcK data is over an 0.8 true negatives rate. This means that for every malicious variant that MimickAV classifies as benign, 80% will also be classified as benign by the anti-virus, with 1% error. This is even more relevant when the curve reaches 0.05 where, at least, 98% will be successfully mimicked, with 5% error. In the case of the Mix data, these values go a bit lower, but it can still imitate this behaviour for more than 90% with 5% error. In NPck malware the results are similar to the results of Mix on those anti-viruses that are easy to imitate, but they are reduced on those that are harder, especially in Kingsoft, which is the most resistant.

Research question 2 asked about the negative predictive value of MimickAV. MimickAV shows a really strong confidence when it provides a negative prediction, especially relevant on packed malware, where it can predict 98% of variants with 5% error.

### 4.3. Detection by Packers

In order to understand which predictions were more sensitive to the packer system, we leverage the knowledge provided by Yara to identify which packers are more and less resistant to our imitation methodology. This is motivated by the alterations on entropy produced by different packers [9]. Table 4 extends the results of Table 2, considering the results of boosting for the top and bottom anti-viruses, with the packers families and the detections related to them, for both, the packed malware and benign-ware in test.

These results show that ASProtect is the less challenging packer (100% for every anti-virus), while the most resistant is ASPack, whose values are ranged between 75% to 100%. All packer systems, but ASPack have detection probabilities above 90%. Some specific packers like UPX and NET, which have the majority of instances according to Table 1, have an accuracy above 95% (UPX ranges from 97% to 98% and NET form 95% to 97%). Borland, which also has a strong number of instances, ranges from 92% to 97%. The rest of the packers range from 96% to 98%. This suggests that, with the exception of ASPack, the different packers are not having a strong influence on the selection of entropy profiles for the analysis.

Research question 3 asked about the influence of packers systems on the accuracy. Packers do not affect the accuracy significantly, except for ASPack, as their accuracy is always above 90%.

### 4.4. Anti-Virus Quality

This last part aims to understand which anti-viruses are more and less resistant to the mimicking process. Based on the previous results from Section 4.1, we consider the boosting classifier results to create an anti-virus ranking, based on the detection abilities of each anti-virus engine in the three datasets (see Figure 1, Figure 2 and Figure 3). Under these conditions, Table 5 shows the ranking with the 57 anti-viruses considered in this work, providing for each anti-virus the mean detection between the three datasets.

There are ten anti-viruses whose imitation accuracy is higher than 95%, headed by eGambit, SUPERAntiSpyware, and Ad-Aware. Forty-five anti-viruses have accuracy detection values betweeen 94% and 95% and only two (ViRobot and Kingsoft) have an accuracy detection under the 93%. Based on these results, and the features that we have used, it is reasonable to consider two main outcomes from them. First, entropy profiles can provide rich information to learn about how anti-viruses are working and, although anti-viruses might not be using entropy profiles directly for detecting malware, they are using features which are, in some way, correlated with the entropy profiles. Otherwise, these imitation rates would be lower. The second conclusion that we can extract from these results is that current anti-virus engines might share information. This can be intuited by analysing, on one hand, the 45 anti-virus programs whose detection rates lie between 94% and 95% of Table 5 (these detection values are extremely similar), and, on the other hand, the detection tendencies of the top 4 classifiers of Figure 1, Figure 2 and Figure 3, where they grow and decrease similarly at different moments, depending on the anti-viruses.    

Research question 4 asked about the accuracy of the most and less resistant anti-viruses. Although it is clear from the accuracy results that eGambit, SUPERAntiSpyware, and Ad-Aware are the most sensitive anti-viruses and ViRobot and Kingsoft the most resistant, our results show that some anti-viruses might share information, as their behaviours are similar according to the top classifiers.

## 5. Related Work

Malware detection techniques have been widely studied in the literature. Section 5.1 provides an overview on the malware arms race. It describes different malware detection techniques based on both static and dynamic analysis, providing some details about different architectures and evasion techniques. Section 5.2 focuses on the application of entropy to malware detection, while Section 5.3 focuses on the importance of anti-viruses research, paying special attention to VirusTotal.

### 5.1. The Malware Arms Race

Malware detection is one of the main sides on the malware arms race. Every time white hats take a step forward, as a reaction to different malicious attacks, black hats find a way to evade this defense, producing a competitive co-evolution between both sides [9].

The main state of the art detection strategies for malware detection are based on static and dynamic analysis. Related to our scenario, Windows malware analysis, there are several methods based on static analysis, like [35], where Santos el at. use disassembled sequences of code, or Opcodes, to discriminate malware from benign-ware, or [36], where Martin et al. use third party library calls to detect invariants for malicious behaviours. From the dynamic analysis perspective, there are several tools that perform traces analysis, such as [37,38] which are based on Cuckoo [4], a virtualization system that extract traces from malware. Similar systems use modifications of QEMU [39] with the same aim [40]. There are also extensions of dynamic analysis that are specifically designed to deal with packers, such as [41].

In different architectures, or types of malware, there are also relevant examples of tools and analysis methodologies. The most populars are those analysing PDF [42], JavaScript [43] and Android [44] malware. Android malware itself is becoming extremely relevant these days [45], and lots of researchers are contributing to this field with several new analysis and detection tools [46,47]. From a static perspective, there are examples like FlowDroid [48], designed to analysise data flows. From dynamic analysis, there are examples like CopperDroid [49], which can analyse traces embedding the Android application into its own virtualization.

According to the most popular detection mechanism, the current state of the art is focused on machine learning techniques [50]. Several examples leverage the information extracted by the previous tools to obtain software features, in order to use them for distinguishing malware from benign-ware [51]. From the static analysis perspective, these features can either feed a classification algorithm (like those described in Section 3.4), using imports [36], opcodes [35], or byte-code n-grams [52]. There are also tools like RevealDroid combining several of these features [53]. From dynamic analysis, the classifiers can also use traces [54], registers [55], or sequences of system calls [56] to obtain high detection accuracy. Machine learning also allows to easily generate hybrid models by combining these features [57].

Nevertheless, there are currently several works proving that machine learning is weak in front of adversaries [50]. The main problem where this sensitiveness relies is the hypothesis that the train and test probability distributions must be equal. Although this assumption has shown its validity with malware in the wild [51], it fails when an adversary has knowledge either on the oracle that decides about the malicious nature of the software, the features chosen for the discrimination process, or the trained model [50]. The work of Biggio and others [58] has shown several examples on how these techniques can be easily defeated. Besides, in the malware detection field, tools such as EvadeML [59], IagoDroid [5], or EEE [9] have proved that those features representing the malware can help to learn how to create undetectable variants.

Current studies are trying to deal with this evasion problem from different perspectives. The works of Goodfellow et al. [60] try to improve the capabilities of classifiers by detecting the adversarial attack directly. Other works leverage game theory, defining a reactive game between the attacker and the defender, and trying to measure its Nash equilibrium [61]. This helps to understand the limitations of the classifiers. However, defending machine learning algorithms from adversaries is still an open problem that requires further investigation.

### 5.2. Entropy on Malware Detection

Entropy has served as one of the main detection metrics for malware for, at least, the last ten years [62]. Originally, it was able to obtain good prediction results, especially after the application of compression or encryption mechanisms for concealing the malware from anti-viruses. The mechanisms left a clear entropy signature that made them detectable [62]. However, as an aggregated measure, it is easily evaded [9], therefore, several authors have either tried to complement it with other metrics based on information theory, such as the normalized compression distance [63], or have given it a different nature, such as the work of Sorokin on structural entropy [8].

Sorokin’s work inspires this paper. On structural entropy, the binary is also divided into fixed size chunks. Nevertheless, the comparison between files is based on a segmentation process that summarizes the information of all the chunks sequence. This summarization is less precise than considering the whole entropy signature or profile, as our previous work has shown [9]. As we require as much information as possible for our micmicking process, we maintained our previous decision of leveraging the whole entropy profile [9]. It is also important to remark that, although entropy has proven to accurately imitate anti-virus behaviours (Section 4.4), we have evidence from our previous work that entropy can be manipulated to defeat classifiers [9], therefore, our future work will measure the influence of these manipulations on real anti-viruses.

### 5.3. Anti-Viruses and VirusTotal

Anti-virus software has evolved in the last years. Originally, it was based on signature-based detection on the binary file, but polymorphism and metamorphism force it to consider other features in order to extend this detection and make it more accurate [64]. Besides, several anti-virus engines have unpacking tools in order to detect malware hidden by known packers. Nevertheless, this technology still relies on signatures, as it is the most precise method in terms of reducing false positives, producing huge datsets of malware signatures. Currently, there are several projects aiming to enrich the anti-virus information with extra analysis, in order to mitigate malware, such as VirusTotal [6].

VirusTotal has helped several researchers to obtain a ground truth on malware detection [65]. This platform provides rich information about the malware binaries, apart of its detection, providing a rich analysis surface. Some works have also leveraged VirusTotal not only to provide the detection, but also to obtain information about the malware families. A good example is AVClass [66]. VirusTotal has also been used to test anti-viruses quality. A good example is Mystique [67]. This auditing tool generates malware variants based on different attack and evasion features. These variants are evaluated against VirusTotal and other anti-malware systems to show their sensitiveness to simple changes. Similarly, DroidChameleon [68] shows how simple and trivial transformation significantly affect the performance of anti-viruses. This was also recently remarked on the work of Hammad et al. [69] that focused these transformation on code obfuscations, discovering that they significantly affect the performance of the anti-viruses. However, at the moment, no work has been focused on trying to imitate the individual anti-viruses behaviour. It is also important to remark that VirusTotal has limitations related to unification of criteria—especially for families [66], and deepness of the analysis. For this reason, we have used malware that is, at least, two years old and has higher probability to be on the VirusTotal dataset, since it comes from a public repository (VirusShare).

## 6. Conclusions and Future Work

Understanding the decisions performed by different anti-viruses is reachable by combining entropy-based features and machine learning, as our tool, MimickAV, has proven. We have shown that for 57 anti-virus engines, MimickAV is able to imitate their behaviour with high accuracy, reaching up to 98% accuracy. It also provides especially good results when it applies a classifier based on boosting. We have also proven that even when the malware is concealed by different packers, which normally alters the entropy of the binary itself, the detection rate is not reduced. Our experimentation has shown that these vulnerabilities might be a consequence of the shared information between different anti-virus companies, as several of them produce similar imitation patterns during the analysis.

According to our results, MimickAV has different auditing applications. From the attacker’s perspective, this similarity between the machine learning model and the anti-virus reduces the needs to check whether a new piece of malware is malicious by using an anti-virus. Therefore, MimickAV reduces the risk of sending the malicious software to the anti-virus company that can perform a deeper analysis finding indicators of compromise. Besides, it is possible that specific modifications to the software that reduce the detection probability of MimickAV might also reduce the detection probability of the specific anti-virus engine, for instance, specific obfuscations that have proven to reduce the detection abilities on anti-viruses [69]. From the defenders perspective, MimickAV shows that anti-viruses are predictable using machine learning methods combined with entropy profiles, and it is relevant to reduce this prediction probability to make sure that they can still contribute to protect computers. Adding more features to the anti-virus prediction, probably extracted from dynamic and static analysis, will decrease this dependency on entropy, which only uses information of the binary itself. This is extremely relevant as the detection of disk resident malware has to be performed before the malware has enough time to infect the system. White hats can leverage MimickAV as a “fitness function”, i.e., as a method to help them to make anti-virus software less predictable, with the aim of defeating this technique, improving the quality of their engines.

Our future work will focus on extending these ideas to the malware families prediction, and measuring how this information can impact on real anti-viruses. We aim to create variants that can defeat these classifiers independently, in order to measure how the strongest ones affect the anti-viruses. This will provide stronger intuition about the importance of our achievements not only with information in the wild, but also with adversarial intentions.

## Figures and Tables

**Table 1:** Information about packers for the packed malware and benign-ware, divided by the number of instances per set.

Packer	Malware	Benign-Ware
Armadillo	542	101
ASPack	186	32
ASProtect	54	10
Borland	2123	417
NET	2351	476
PECompact	445	88
UPX	3175	641
Rest	1124	235
Total	10,000	2000

**Table 2:** Accuracy results for the best and worst classifiers and anti-viruses.

Model	Top 2 MimickAV		Bottom 2 MimickAV	
Pck boosting	SASpyware (97.8)	TotalDefense (97.8)	Endgame (96.2)	McAfee (96.2)
NPck boosting	eGambit (96.2)	ALYac (94.7)	ViRobot (86.4)	Kingsoft (85.0)
Mix boosting	eGambit (96.2)	TheHacker (95.7)	Kaspersky (93.0)	Endgame (92.9)
Pck ada	SASpyware (97.3)	TotalDefense (97.1)	K7GW (95.5)	Cyren (95.5)
NPck ada	eGambit (95.1)	Ad-Aware (94.0)	ViRobot (86.3)	Kingsoft (84.9)
Mix ada	TheHacker (95.3)	eGambit (95.2)	Avast (92.0)	CrowdStrike (91.9)
Pck h2o.gbm	TotalDefense (97.1)	SASpyware (97.0)	K7GW (95.4)	Cyren (95.4)
NPck h2o.gbm	eGambit (95.1)	ALYac (93.7)	ViRobot (86.4)	Kingsoft (84.7)
Mix h2o.gbm	TheHacker (95.2)	eGambit (95.0)	Avast (91.9)	Endgame (91.8)
Pck OneR	AhnLab-V3 (88.7)	Kaspersky (88.5)	GData (86.0)	MAX (86.0)
NPck OneR	ALYac (89.1)	eGambit (88.5)	TrendMicro (80.1)	Kingsoft (78.6)
Mix OneR	Ad-Aware (86.3)	ALYac (85.5)	Bkav (80.3)	TMHouseC (79.6)
Pck nnet	Rising (91.5)	AhnLab-V3 (91.4)	ALYac (86.2)	NANO (85.6)
NPck nnet	eGambit (88.2)	ALYac (88.2)	Bkav (77.7)	ViRobot (77.7)
Mix nnet	Baidu (87.3)	eGambit (87.1)	TMHouseC (81.9)	Kingsoft (81.4)
Pck NBayes	Panda (84.4)	TotalDefense (84.1)	Paloalto (80.2)	ENOD32 (79.7)
NPck NBayes	TMHouseC (79.7)	TheHacker (78.7)	Kingsoft (72.0)	SASpyware (71.4)
Mix NBayes	CMC (86.5)	TrendMicro (82.9)	GData (77.3)	SASpyware (76.4)

**Table 3:** False negatives and true negatives rates for the best and worst anti-virus (AV) predictions of boosting. Bold characters highlight the best results.

TNR/FNR	0	0.002	0.01	0.05	0.1	0.15
Pck SASpyware	0.04	0.11	**0.93**	**0.99**	**1.0**	**1.0**
Pck TotalDefense	0.05	0.26	0.80	**0.99**	**1.0**	**1.0**
Pck Endgame	0.01	0.03	**0.93**	0.98	0.99	**1.0**
Pck McAfee	**0.20**	**0.64**	0.81	**0.99**	**1.0**	**1.0**
NPck eGambit	**0.80**	**0.83**	**0.88**	**0.96**	**0.98**	**0.98**
NPck ALYac	0.06	0.26	0.64	0.94	0.97	**0.98**
NPck ViRobot	0.18	0.24	0.48	0.73	0.80	0.88
NPck Kingsoft	0.09	0.12	0.25	0.54	0.72	0.82
Mix eGambit	0.08	0.14	**0.79**	**0.96**	**0.98**	**0.99**
Mix TheHacker	0.01	0.13	0.58	0.91	0.97	0.98
Mix Kaspersky	**0.13**	**0.51**	0.60	0.94	0.97	**0.99**
Mix Endgame	0.11	0.19	0.68	0.92	0.96	0.98

**Table 4:** Breakdown of boosting in Table 2, divided into packing systems of Pck. Bold characters highlight the best results, in this case, results higher than 99%.

Packer	SASpyware	TotalDefense	Endgame	McAfee
Armadillo	97.30%	94.87%	95.12%	96.88%
ASPack	75.00%	**100.0**%	80.00%	93.33%
ASProtect	**100.0**%	**100.0**%	**100.0**%	**100.0**%
Borland	97.20%	93.66%	92.16%	94.48%
NET	97.96%	96.51%	95.49%	96.64%
PEComp	**100.0**%	**100.0**%	93.55%	97.30%
UPX	98.22%	97.98%	97.69%	97.35%
Rest	98.43%	98.22%	96.23%	98.09%

**Table 5:** Whole anti-virus ranking after applying MimickAV with boosting. These results show the mean of the three datasets.

Anti-Virus	Accuracy	Anti-Virus	Accuracy	Anti-Virus	Accuracy
eGambit	96.50%	TotalDefense	94.74%	K7GW	94.48%
SASpyware	95.75%	CATQHeal	94.74%	Comodo	94.44%
Ad-Aware	95.65%	K7AntiVirus	94.71%	McAfee-GW	94.44%
ALYac	95.53%	Cyren	94.70%	TheHacker	94.41%
Rising	95.40%	BitDefender	94.68%	Symantec	94.38%
F-Prot	95.36%	DrWeb	94.68%	MAX	94.38%
Jiangmin	95.33%	Microsoft	94.65%	Avira	94.34%
ClamAV	95.21%	Invincea	94.64%	Avast	94.33%
AhnLab-V3	95.15%	SentinelOne	94.64%	Paloalto	94.33%
Bkav	95.00%	ZoneAlarm	94.60%	Kaspersky	94.33%
Webroot	94.92%	Qihoo-360	94.60%	Fortinet	94.30%
MicroWorld	94.90%	Emsisoft	94.60%	NANO	94.24%
Baidu	94.89%	TrendMicro	94.59%	AVware	94.24%
Tencent	94.85%	CrowdStrike	94.59%	ENOD32	94.23%
GData	94.82%	F-Secure	94.59%	VIPRE	94.20%
Arcabit	94.79%	VBA32	94.59%	Endgame	94.13%
Sophos	94.77%	CMC	94.57%	McAfee	94.07%
Panda	94.77%	AVG	94.52%	ViRobot	92.70%
Yandex	94.74%	TMHouseC	94.50%	Kingsoft	92.30%
